# Metal-Multilayered Nanomechanical Cantilever Sensor for Detection of Molecular Adsorption

**DOI:** 10.3390/bios13060573

**Published:** 2023-05-23

**Authors:** Masaya Toda, Takahito Ono, Jun Okubo

**Affiliations:** Department of Mechanical Systems Engineering, Tohoku University, Sendai 980-8579, Japan

**Keywords:** MEMS, adsorption, stress sensor

## Abstract

A metal-multilayered nanomechanical cantilever sensor was proposed to reduce the temperature effect for highly sensitive gas molecular detection. The multilayer structure of the sensor reduces the bimetallic effect, allowing for the detection of differences in molecular adsorption properties on various metal surfaces with higher sensitivity. Our results indicate that the sensor exhibits higher sensitivity to molecules with greater polarity under mixed conditions with nitrogen gas. We demonstrate that stress changes caused by differences in molecular adsorption on different metal surfaces can be detected and that this approach could be used to develop a gas sensor with selectivity for specific gas species.

## 1. Introduction

Medical costs have been increasing in recent years due to the aging of society and the rise of lifestyle-related diseases. To reduce these costs and alleviate the burden on medical personnel, there is a growing need for breathalyzer devices that can predict diseases in advance [1]. It is well-established that exhaled breath contains gas components, and clinical studies have shown that certain volatile organic compounds (VOCs) are related to various diseases [2,3,4]. For example, acetone, a VOC found in exhaled breath, is associated with diabetes and could be used as a biomarker for diabetes diagnosis. However, due to the fact that breath is a mixture of various gases, including water, and that changes in the concentration of gas components occur at very low levels (ppm or lower ppb), breathalyzer diagnosis is not yet feasible using current sensor sensitivity and gas species selectivity. This study proposes a gas sensor for breathalyzer diagnosis that utilizes the difference in molecular adsorption characteristics on different metal surfaces. Conventional gas sensors use a two-layer structure with a film deposited on only one side of the sensor that adsorbs specific gas molecules [5,6,7].

Cantilever-type stress sensors typically detect physical deformation resulting from differences in surface stress caused by variations in the adsorption state of the gas being analyzed on the sensing film and cantilever [8,9,10]. A model based on atomic or molecular interaction was discussed for adsorption-induced surface stress [11]. The model was considered by Hg adsorption on Au-coated cantilevers, providing insight into the interatomic forces that play a significant role in creating adsorption-induced surface stresses and the resulting mechanical bending of cantilevers. The deflection of the cantilever can be detected either optically using an optical reflecting method or through a change in resistance using the piezoresistive effect [12,13,14]. The piezoresistive effect is a phenomenon whereby the electrical resistance of a material changes when stress is applied [15]. Although the optical method for detecting displacement is relatively more complex than the method using piezoresistive elements, it has been shown to yield a detection sensitivity two orders of magnitude higher. To increase the sensitivity of the sensor, a longer structure is required, but fabricating such a structure is difficult due to deformation caused by thermal residual stress, which arises in structures combining different materials. If such a long structure has been successfully developed, the achievable sensitivity will open up the next stage of diagnostic applications. For a bilayer cantilever with a sandwich structure, small temperature changes can cause deflection due to differences in thermal expansion between the layers, which is commonly known as the “bimaterial effect” [16,17,18]. This effect can also induce thermal residual stress during the fabrication process [19]. 

**Figure 1:**
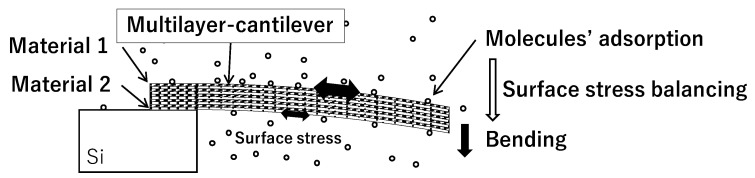
Measurement principle of the metal-multilayered nanomechanical cantilever sensor.

In order to tackle the problem of deformation caused by residual thermal stress in structures that combine different materials, there is a need for innovative solutions that can address this challenge. One potential solution is the use of a multilayer structure that incorporates two different types of metals, each of which can create a different surface on the top and bottom sides of the structure [20,21]. This approach, as depicted in Figure 1, has shown promising results in mitigating the effects of thermal stress. Typically, in the multilayered coatings on the microcantilever, the thermal stress has a strong temperature dependence in bending [22,23]. The neutral plane can be set in the multilayers to build the multilayered films as a thin cantilever with a substrate. To obtain deflection due to molecular adsorption, traditional cantilever structures require at least two metals with different surfaces on the top and bottom, which results in a significant bimaterial effect [24]. In our proposed multilayered structure, we expect that the bimaterial effect will be effectively reduced. The surface energy change caused by molecular adsorption can result in surface stress, which may indirectly contribute to thermal stress by altering the material’s thermal properties. This can cause uneven heating and cooling of the material, leading to bending or deformation. In a multilayered structure, the purpose is to minimize the heating effect and reduce the bending of the cantilever, making it less sensitive to temperature changes and allowing for direct deflection caused by molecular adsorption to be observed. The multilayer structure approach not only addresses the deformation caused by residual thermal stress in structures combining different materials but also provides several additional benefits. One of the key advantages of this approach is that it enables the fabrication of longer structures than those that can be made with traditional single-material structures. This is due to the increased ability of the multilayer structure to distribute stress more evenly, reducing the risk of deformation and damage over longer lengths. Our research also focuses on multi-component sensing, which requires a simple and efficient functionalization process for the sensor array. While methods such as polymer coating and absorber layers with nanoparticles have been shown to be effective, they are more complex in terms of functionalization [25,26]. Therefore, traditional deposition methods using simple metal surfaces are promising approaches for functionalization, which offer the added benefit of avoiding issues related to sensitivity. The effectiveness of the multilayer structure approach in improving sensitivity to stress resulting from molecular adsorption has been demonstrated through the successful fabrication and evaluation of a molecular adsorption stress detection sensor. This sensor is composed of a multilayer metal film that has been specifically designed to exhibit high sensitivity and selectivity for breathalyzer diagnosis.

## 2. Designing of the Multilayered Structure

Adsorption is a complex process that involves the interaction between an adsorbent surface and an adsorbate. The adsorbate can be any molecule, ion, or atom that is attracted to the adsorbent surface, and the strength of this interaction depends on a number of factors, including the properties of the adsorbate and adsorbent surfaces, as well as the environmental conditions. Adsorption can be classified into two types: physical adsorption and chemisorption. Physical adsorption is a weak interaction that occurs as a result of van der Waals forces, dipole–dipole interactions, and hydrogen bonding. It is typically reversible, and the adsorption energy is usually in the range of 1–10 kJ/mol. Physical adsorption is often used in gas separation, chromatography, and catalysts. Chemisorption, on the other hand, is a stronger interaction that involves the formation of chemical bonds between the adsorbate and adsorbent surfaces. It can involve the transfer of electrons between the two surfaces, resulting in the formation of covalent or ionic bonds. The adsorption energy for chemisorption is typically one or two orders of magnitude higher than that of physical adsorption, and it is often irreversible [27]. Chemisorption is important in catalysis, surface modification, and electronic devices [28]. The surface energy of a material is a measure of the energy required to increase the surface area of the material. It is typically measured in units of mJ/m^2^ and is equivalent to surface stress, which is the force per unit length. Surface energy plays an important role in determining the adsorption behavior of materials, as it influences the strength of the interaction between the adsorbent surface and the adsorbate. Higher surface energy materials tend to have stronger adsorption properties and can be more effective in capturing and separating target molecules [29,30]. 

**Figure 2:**
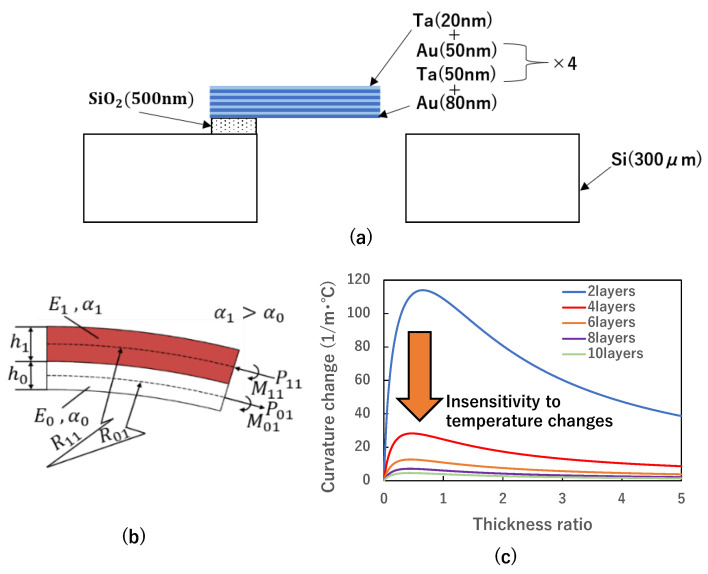
(**a**) Schematic of the proposed device cantilever-type sensor, (**b**) a calculation model of a 2-layer beam, (**c**) comparison of the proposed device with a conventional 2-layer structure.

One of the objectives is to reduce the effect of residual stress caused by the deposition process, while another is to mitigate the bimaterial effect of temperature dependence. In Figure 2a, the proposed cantilever-type gas sensor with a multilayer structure of five bilayers composed of thin gold and tantalum films is depicted. The use of this structure offers a number of advantages over sensors fabricated with polymer and silicon materials.

When a temperature change ∆T occurs in a structure consisting of different materials, elastic forces are generated internally to compensate for the differences in the coefficients of thermal linear expansion, resulting in the curvature of the structure, as illustrated in Figure 2b. We consider a multilayered structure composed of two materials, where the strain at the bonding surface is equal and can be expressed by the following Equation (1), given that the width of the beam is *b*.

(1) $$\alpha_{1}\Delta T-\frac{P_{11}}{bh_{1}E_{1}}-\frac{h_{1}}{2R_{11}}=\alpha_{0}\Delta T+\frac{P_{01}}{bh_{0}E_{0}}+\frac{h_{0}}{2R_{01}}\;$$

Here, $\alpha_{0}$ and $\alpha_{1}$ represent the expansion coefficient of each layer, $R_{01}$ and $R_{11}$ represent the radius of curvature, $h_{0}$ and $h_{1}$ represents the thickness of each layer, $E_{0}$ and $E_{1}$ represent Young’s moduli of each layer, and $P_{01}$ and $P_{11}$ represent the lateral forces acting on each layer.

On the other hand, the bending moments $M_{11}$ and $M_{01}$ are required to curve into an arc of the radius of curvature $R_{11}$ and $R_{01}$ are the sectional secondary moment $I_{0}$ and $I_{1}$, respectively.

(2) $$M_{11}=\frac{E_{1}I_{1}}{R_{11}}\;,\;M_{01}=\frac{E_{0}I_{0}}{R_{01}}\;$$

At this time, no force is acting in the horizontal direction, and no moment is acting, so the following two equations are valid.

(3) $$P_{01}-P_{11}=0\;$$

(4) $$M_{11}+M_{01}-\frac{P_{01}h_{0}}{2}-\frac{P_{11}h_{1}}{2}=0$$

Here, if we consider that the thickness of each layer is very small, we can approximate $R_{11}$=$\;R_{01}\;$= R. Under this approximation, Equations (2)–(4) can be solved jointly for curvature 1/*R* as follows.

(5) $$\frac{1}{R}=\frac{6h_{0}h_{1}E_{0}E_{1}\left(\alpha_{1}-\alpha_{0}\right)\left(h_{0}+h_{1}\right)\Delta T}{h_{0}{}^{4}E_{0}{}^{2}+4h_{0}{}^{3}h_{1}E_{0}E_{1}+6h_{0}{}^{2}h_{1}{}^{2}E_{0}E_{1}+4h_{0}h_{1}{}^{3}E_{0}E_{1}+h_{1}{}^{4}E_{1}{}^{2}}\;$$

This can be transformed as follows when $t=h_{0}+h_{1}\;,m=\frac{E_{0}}{E_{1}},\;n=\frac{h_{0}}{h_{1}}$,

(6) $$\frac{1}{R}=\frac{6\left(\alpha_{1}-\alpha_{0}\right)mn{\left(1+n\right)}^{2}\Delta T}{t\left(m^{2}n^{4}+4mn^{3}++6mn^{2}+4mn+1\right)}$$

In a sensor structure that combines dissimilar materials, differences in the linear expansion coefficients of the materials cause residual thermal stress, which deforms the sensor body. 

As shown in Equation (6), the larger the coefficient of linear expansion, the larger the deformation. We propose here to further increase the number of layers as a method to reduce the effect of thermal residual stress. The effect of reducing thermal residual stress by increasing the number of layers can be seen from the theoretical equations of curvature for 4, 6, 8, and 10 layers. The amount of deformation is considered when a temperature change ∆T occurs in a structure made of different materials. Equation (6) shows the change in curvature for the 4-layer case.

(7) $$\frac{1}{R}=\frac{6\left(\alpha_{1}-\alpha_{0}\right)mn{\left(1+n\right)}^{2}\Delta T}{t\left(4m^{2}n^{4}+10mn^{3}+3n^{2}+6m^{2}n^{3}+18mn^{2}+6n+3m^{2}n^{2}+10mn+4\right)}$$

Similarly, for layers 6, 8, and 10, the change in curvature when the temperature of the structure changes by ∆T is obtained as follows:

(8) $$\frac{1}{R}=\frac{6\left(\alpha_{1}-\alpha_{0}\right)mn{\left(1+n\right)}^{2}\Delta T}{t\left(9m^{2}n^{4}+20mn^{3}+8n^{2}+16m^{2}n^{3}+38mn^{2}+16n+8m^{2}n^{2}+20mn+9\right)}$$

(9) $$\frac{1}{R}=\frac{6\left(\alpha_{1}-\alpha_{0}\right)mn{\left(1+n\right)}^{2}\Delta T}{t\left(16m^{2}n^{4}+34mn^{3}+15n^{2}+30m^{2}n^{3}+66mn^{2}+30n+15m^{2}n^{2}+34mn+16\right)}$$

(10) $$\frac{1}{R}=\frac{6\left(\alpha_{1}-\alpha_{0}\right)mn{\left(1+n\right)}^{2}\Delta T}{t\left(25m^{2}n^{4}+52mn^{3}+24n^{2}+48m^{2}n^{3}+102mn^{2}+48n+24m^{2}n^{2}+52mn+25\right)}$$

One advantage is reduced deformation caused by temperature change due to the similar linear expansion coefficients of gold and tantalum. This means that the multilayered structure can better withstand temperature changes without deforming, which is important for accurate and reliable sensing. Another advantage is reduced susceptibility to deformation due to residual thermal stress with an increase in the number of layers. This is because the multilayered structure distributes the thermal stress more evenly across the layers, reducing the overall stress on each individual layer. Increasing the number of layers also enables the fabrication of a longer sensor structure, resulting in higher sensitivity. This is because a longer structure provides a larger surface area for the adsorption of gas molecules, increasing the likelihood of detecting them. To evaluate the effectiveness of the proposed design in reducing susceptibility to deformation due to residual stress, theoretical calculations using Equation (6)–(10) were carried out, as shown in Figure 2c. Here, to evaluate the effect of temperature, the curvature changes of the cantilever per 1 °C were calculated. The temperature dependence affects the performance of deflection caused by molecular adsorption, and a lower change in the curvature is preferred. Gold and tantalum, the selected materials for the sensor, were used in the calculation, with a total film thickness of 0.5 µm. Figure 2c illustrates the calculated curvature change per unit temperature change for the conventional two-layer structure and the proposed sensor structure. The results demonstrate that increasing the number of layers in the multilayered structure reduces the susceptibility to deformation due to residual thermal stress. Specifically, the curvature change per unit temperature change is significantly reduced in the proposed sensor structure compared to the conventional two-layer structure. This means that the multilayered structure is better able to withstand residual thermal stress and maintain its original shape, which is critical for accurate and reliable gas sensing. 

## 3. Device Fabrication

**Figure 3:**
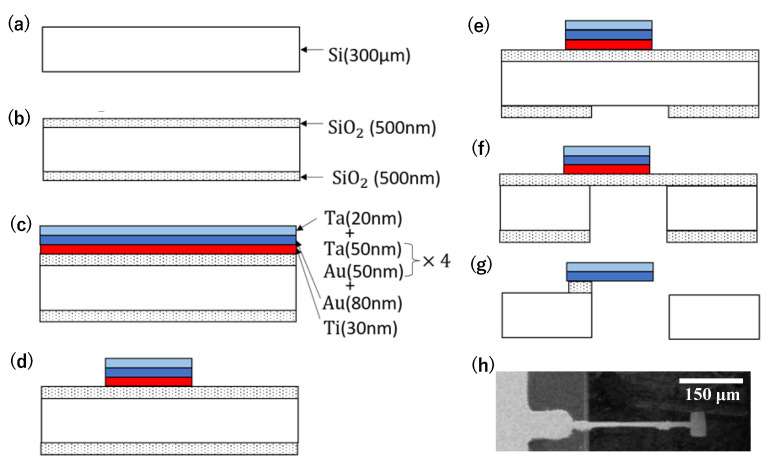
Fabrication process of the sensor device. (**a**) Wafer cleaning, (**b**) deposition of SiO_2_ on both sides, (**c**) metal deposition, (**d**) patterning of metal layers, (**e**) patterning of SiO_2_, (**f**) deep reactive ion etching of Si handling layer, (**g**) wet etching of SiO_2_, and (**h**) completed cantilever SEM image.

The process of fabricating the sensor device is illustrated in Figure 3. First, a 300 µm Si wafer was diced and used to make the sensor device (Figure 3a). Next, a 500 nm SiO_2_ layer was deposited on both sides of the wafer using plasma-enhanced chemical vapor deposition (PECVD) with tetraethyl-orthosilicate (TEOS), as shown in Figure 3b. The deposition rate was approximately 83 nm/min under the process conditions of 80 Pa pressure and 300 °C temperature. Then, 30 nm of Ti, Au, and Ta were sputtered onto the SiO_2_ layer on the surface to create a total thickness of 500 nm, as shown in Figure 3c. After patterning by photolithography, the metal layers were etched by ion beam milling, as shown in Figure 3d. The etching rate was between 20 nm and 30 nm for each metal layer. The SiO_2_ layer on the back side was patterned using buffered hydrofluoric acid (BHF), as shown in Figure 3e. The handling Si was then etched using a deep-reactive ion etching (RIE) process, as shown in Figure 3f. The standard recipe with the etching rate of 2.5 μm/min was used. Finally, the insulative SiO_2_ layer was etched with BHF to release the structure, and the sensor device was completed by supercritical CO_2_ drying (Figure 3g). The actual fabricated sensor device is depicted in Figure 3h. The dimensions of the cantilever beam used in this example are 600 µm in length, 25 µm in width, and 0.5 µm in thickness.

## 4. Measurement and Results

### 4.1. Surface Profile

**Figure 4:**
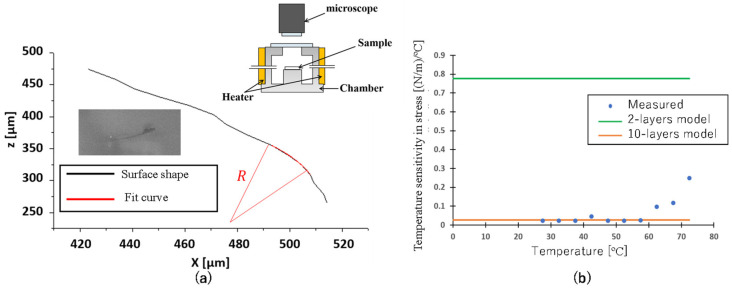
(**a**) Evaluation setup for cantilever-type sensors and fitting curve to determine the radius of the beam. (**b**) the temperature sensitivity in stress calculated by the obtained radius as the responses to temperature change with theoretical values for the 2 and 10-layer models.

The surface profile of the fabricated cantilever sensor was observed from the side using a microscope, as shown in Figure 4a. To measure the deformation of the cantilever, we changed the temperature of the sensor using a hot plate and observed the resulting bending [31]. Placing the sample on the hotplate under the microscope, the temperature was manually changed step by step using a 10× objective for measurement. Hundreds of points were then traced from the resulting image to obtain a fitted profile curve using a polygonal line. This allowed for a detailed analysis of the surface profile of the cantilever. During this analysis, it was observed that the cantilever was warped downwards, indicating a deformation in the structure. The curvature of the cantilever was then obtained by fitting the longitudinal shape of the sensor obtained from the surface profile measurement. 

### 4.2. Temperature Dependence

To assess the ability of the cantilever-type sensor to resist deformation induced by temperature changes, the curvature stability was tested by varying the sample temperature. The temperature range was set from 25 °C to 75 °C in increments of 5 °C using a rubber-type ribbon heater. This temperature range was selected because it falls within the safe operating range of the microscope system. It is also sufficient for observing the temperature dependence of the curvature change. At each temperature, the surface profile of the sensor was measured after waiting for the temperature, usually in 10 min, and the curvature was obtained by fitting. The obtained curvature data was then used to calculate the surface stress per unit temperature, and the results were plotted in Figure 4b. The cantilever-type sensor used for this evaluation had dimensions of 600 µm in length, 0.5 µm in thickness, and 25 µm in width. In the temperature range below 60 °C, the values generally agreed with the theoretical values for the 10-layer sensor, although they were slightly smaller. At temperatures above 60 °C, the surface stress sensitivity increased, possibly due to the buckling of the sensor caused by thermal stress. These results demonstrate that the proposed design can effectively reduce the susceptibility of the sensor to deformation caused by residual thermal stress. The use of multiple layers in the sensor structure provides robust mechanical support, allowing the sensor to maintain its sensitivity to surface stress even at higher temperatures. Compared to a 2-layer model, which is a traditional structure for a bimaterial cantilever to have a different surface on top and bottom, the measurement of temperature sensitivity is still a priority for the multilayer cantilever. By changing the temperature, the adsorption model for gas sensing provides more options as a variable parameter to determine the gas components. The wide range of operating temperatures is also a good point for sensing applications. 

### 4.3. Response to Gas/Vapor

**Figure 5:**
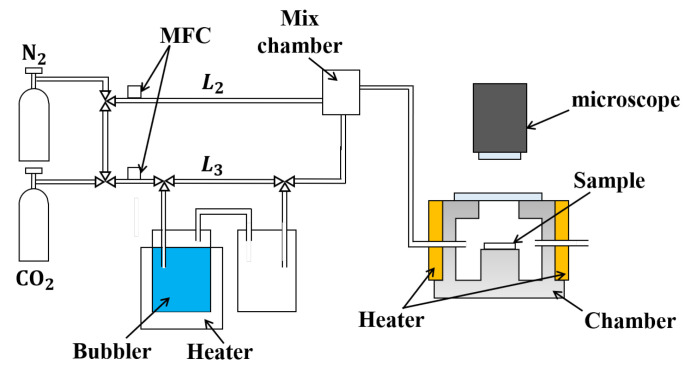
Experimental setup for response to controlled test gasses.

The experimental setup for measuring the response of the cantilever-type sensor to water, acetone vapors, and CO_2_ gas is depicted in Figure 5. The sample was installed into the sample chamber. This metallic sample chamber has a volume of approximately 0.001 m^3^. A rubber ribbon heater was employed to maintain a constant temperature of 25 °C, which was controlled using a PID system. The gas mixture was prepared using a homemade gas flow system, which employed two mass flow controllers (MFCs). The different gas components were mixed with dry gas to achieve the desired concentration and saturation levels by bubbling. N_2_ gas continuously flowed into the gas chamber, and N_2_ gas continuously flowed into the gas chamber at a rate of 500 sccm for 20 min, which is the usual stabilizing time in the sample chamber. A mixing chamber was utilized to ensure that the saturated vapor and dry N_2_ gas were well mixed. During the flowing experiment, water vapor, acetone vapor, and diluted CO_2_ were introduced into the sample chamber at the same flow rate of 500 sccm. In this report, we focus on the partial pressure of the sensing element, which is more theoretically analyzable with absolute concentration. The deflection of the sensor was observed immediately, and after waiting for the stabilized motion, the surface profiles of the sensor were examined before, during, and after exposure under dry conditions. The experiment was carried out on two sensors, both of which had dimensions of 300 µm in length, 0.5 µm in thickness, and 25 µm in width. The results were averaged to obtain a more accurate representation of the sensor’s response.

**Figure 6:**
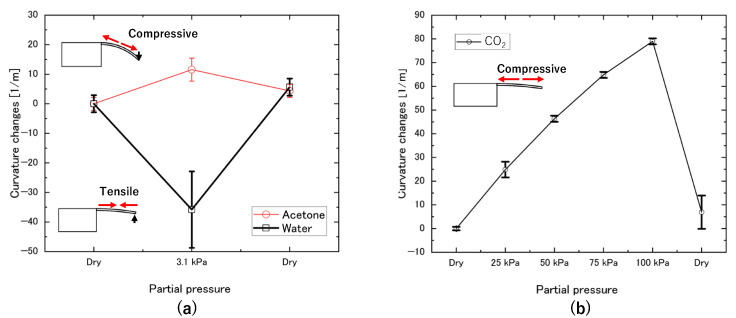
(**a**) Curvature variations against water and acetone vapors at a partial pressure of 3.1 kPa, (**b**) curvature variation against CO_2_ with varying partial pressure.

The plots of curvature variation versus water vapor and acetone vapor at a partial pressure of 3.1 kPa are shown in Figure 6a. The partial pressure of 3.1 kPa of water vapor indicates the saturated water vapor under 25 °C. The flow rate of L_3_ was set to 500 sccm. Three sets of experiments were conducted for each type of gas using two different cantilevers of different sizes to ensure the reproducibility of the results. To perform statistical analysis, we calculated the mean and standard deviation of the measurements obtained in each set of experiments. It can be observed that the curvature of the sensor decreased and saturated after 20 min due to the flow of water vapor into the gas chamber, which caused tensile stress on the surface of the sensor. This effect is believed to be caused by the aggregation of water molecules on the surface through hydrogen bonding. 

The response of the sensor to acetone vapor was evaluated by adjusting the flow rates of L_2_ and L_3_ to 437 sccm and 63 sccm, respectively, to achieve a partial pressure of acetone in the chamber equal to the saturation vapor pressure of water at 25 °C (3.1 kPa). The temperature was maintained at 25 °C using a heater, and N_2_ gas flowed at a constant rate of 500 sccm for 20 min in the gas chamber. As shown in the inset image in Figure 6a, the curvature increased due to the flow of acetone, indicating that compressive stress was generated on the surface of the sensor. The difference in curvature response between the water and acetone experiments suggests that the sensor can detect differences in molecule adsorption.

The sensor’s response to CO_2_ was evaluated by varying the CO_2_ flow rate to 0, 25, 50, 75, and 100% using a mass flow controller, and the surface profile was observed at each concentration. The obtained curvatures from two sensors, 300 µm long, 0.5 µm thick, and 25 µm wide, were averaged. Figure 6b shows that the curvature increases after exposure to CO_2_, indicating that the sensor experiences compressive stress as in the inset image in Figure 6b. Moreover, the sensor curvature is found to increase with increasing CO_2_ concentration, indicating that the sensor’s response is concentration-dependent.

## 5. Discussion

The bending response of detecting gas concentration in cantilever-type sensors can be determined by calculating the theoretical value for a deflection of 1 nm using optical methods such as laser displacement measurement. Although the thermomechanical vibration is typically less than 1 nm, the environmental fluctuation affects the resolution and detection limit, resulting in a detectable low level just below 1 nm. It is essential to have a high length-to-thickness ratio for the cantilever-type sensor to increase the sensitivity of detecting surface stress. A higher ratio will result in a greater sensitivity of detecting surface stress when the tip of the sensor deflects, which plays a critical role in determining the bending response of the cantilever-type sensor for detecting gas concentration.

We adjusted the partial pressures of each gas to the same value by changing the flow speed, making the absolute concentrations comparable among the different gas species in our experiments. Therefore, we believe that our experiments show statistically reliable results. Table 1 summarizes the curvature changes for each gas partial pressure of 3.1 kPa. The results of the adsorption experiments for water, acetone, and carbon dioxide gases indicate that the amount of curvature change differs between positive and negative dynamics, respectively, likely due to the influence of polarity and characteristic length on the ease of adsorption. This suggests the potential for gas species discrimination. Assuming a cantilever length of 1000 µm, we can estimate the actual measurements required for a 1 nm displacement of the tip. However, the actual bending deflections were 36–92 times worse than the expected deflections. The reduced bending response of the cantilever-type sensor when detecting gas concentration can be attributed to the occurrence of physical adsorption on both surfaces of the sensor. This phenomenon is caused by weak electrical interactions such as van der Waals forces, which result in the target molecules being adsorbed on both the bottom and top surfaces of the sensor. This leads to a reduction in sensitivity due to the small difference in adsorption amounts.

**Figure 7:**
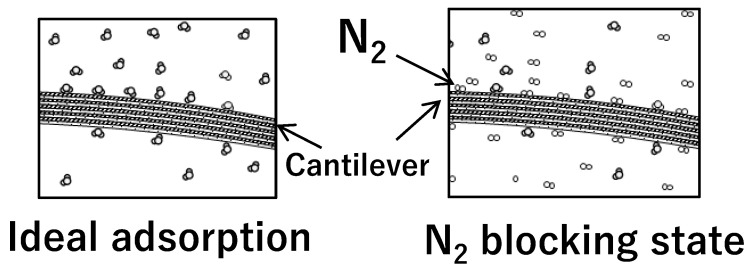
Nitrogen molecules are not present on the surface adsorption site (**left**) or are present on the surface adsorption site (**right**).

In the gas response experiment, nitrogen gas first flowed into the chamber to establish a baseline, and then the sample gas was introduced to measure the response. This allows for a comparison of the adsorption of the target gas molecules to the baseline adsorption of the nitrogen gas. By subtracting the baseline response from the sample response, the specific response to the target gas can be determined. The measurement of this specific response can be used to determine the concentration of the target gas. Figure 7 shows that an effective amount of nitrogen could already be adsorbed on the tantalum surface. This adsorption of nitrogen at the adsorption site may reduce the coverage of the sample gas in the saturated state, leading to a decrease in the sensitivity of the sensor. Additionally, the higher the polarity of the molecule, the greater the bending response. The response obtained in the experiment is considered to be influenced by the relative ease of adsorption compared to nitrogen. The fact that the bending response of the sensor increases with polarity suggests that the ease of adsorption is affected by polarity, resulting in greater adsorption. For practical applications such as breath diagnosis, the gas mixture contains nitrogen gas, which can affect the bending response due to the specific gas concentration. Therefore, it is important to consider the theoretical estimation of nitrogen gas in future gas-sensing research.

## 6. Conclusions

A multilayered structure using two types of metals was proposed for the stress sensor to achieve high sensitivity and stability in detecting molecular adsorption. This multilayered structure is designed to reduce deformation caused by residual thermal stress, enable longer lengths, and improve sensitivity to stress due to molecular adsorption. Experiments were conducted to evaluate the sensor’s tendency to deform in response to changes in temperature, and the results showed that the sensor deformation was almost the same as the theoretical value at temperatures below 60 °C and that the amount of deformation was reduced. These findings suggest that the multilayered structure could effectively minimize thermal stress-induced deformation, which is essential for accurate and reliable stress sensing. Additionally, the use of two metals in the structure can provide a larger difference in adsorption energy, which can enhance the sensor’s bending response to molecular adsorption.

Based on the gas response of the sensor, the estimated sensitivities of the sensor at a length of 1000 μm were 1.8 ppm/nm for water, 5.6 ppm/nm for acetone, and 11 ppm/nm for CO_2_. In all experiments, the curvature did not return to its original value, likely due to the physical blocking of molecular desorption and adsorption on the sensor surface. These values are two orders of magnitude smaller than the theoretical values, possibly due to smaller differences in adsorption caused by physical adsorption. The sensor’s bending response was found to be higher for molecules with greater polarity, likely because polarity affects adsorption, resulting in increased adsorption. 

In this study, we fabricated and evaluated a molecular adsorption stress sensor with a multilayer structure for gas sensing, utilizing the different molecular adsorption properties on various metal surfaces. Polarity and adsorption have a complex relationship that depends on various factors. Along with polarity, additional variables that can influence the adsorption of polar molecules onto a metal layer include the surface area and roughness of the metal layer, as well as temperature and pressure. If the metal layer is rougher and has a larger surface area, it can offer more surfaces for polar molecules to adsorb on, thereby increasing the sensitivity of the sensor. Environmental factors, metal surface properties, and adsorbing molecule characteristics can affect stress changes induced by molecular adsorption on metal layers. Metal layer characteristics such as crystallographic orientation, thickness, and the presence of multiple crystals can also impact stress changes. Additionally, defects such as dislocations and vacancies can affect stress changes as well. Our results demonstrated that the stress changes caused by differences in molecular adsorption on different metal surfaces could produce a response, indicating that a selective stress sensor could be developed for detecting specific gas species. Another potential avenue for improvement is to optimize the metal layers used in the sensor’s multilayered structure. For example, different metals or metal alloys could be used to enhance the selectivity of the sensor for specific gas molecules or to improve its overall sensitivity. In addition, the thickness and composition of the metal layers could be varied to tune the sensor’s response to different gases or to optimize its mechanical stability. Alternatively, the stress sensor could be combined with a surface plasmon resonance sensor or a quartz crystal microbalance sensor to create a hybrid sensing system that utilizes multiple sensing modalities to improve its overall performance.

## Figures and Tables

**Table 1:** The bending response of the sensor to each gas and the theoretical value for each gas partial pressure of 3.1 kPa and sensor length of 1000 μm with varying curvature.

	Curvature Change	Curvature Change_exp_	Curvature Change_cal_
Water	−36 ± 13 m^−1^	1.8 ppm/nm	50 ppb/nm
Acetone	11± 4.0 m^−1^	5.6 ppm/nm	100 ppb/nm
CO_2_	6.1 ± 1.1 m^−1^	11 ppm/nm	12 ppb/nm
